# Squamous cell carcinoma is associated with reduced IL34 expression, alterations in the Langerhans cell antigen‐processing‐presentation machinery and poor patient survival

**DOI:** 10.1002/cti2.70018

**Published:** 2024-11-29

**Authors:** Thi Viet Trinh Dang, Kevin R Gillinder, Quan Nguyen, Onkar Mulay, Tuan Vo, Ahmed M Mehdi, Chenhao Zhou, Andrew J Brooks, Graham R Leggatt, David A Hume, Ian H Frazer, Janin Chandra

**Affiliations:** ^1^ Faculty of Medicine, Frazer Institute The University of Queensland Brisbane QLD Australia; ^2^ Institute for Molecular Bioscience The University of Queensland Brisbane QLD Australia; ^3^ Queensland Institute of Medical Research Berghofer Herston QLD Australia; ^4^ School of Biomedical Sciences and Pharmacy The University of Newcastle Callaghan NSW Australia; ^5^ Precision Medicine Research Program Hunter Medical Research Institute New Lambton Heights NSW Australia; ^6^ QCIF Bioinformatics Queensland Cyber Infrastructure Foundation Ltd Brisbane QLD Australia; ^7^ Mater Research Institute The University of Queensland Brisbane QLD Australia

**Keywords:** epithelial hyperplasia, IL34, Langerhans cells, squamous cell carcinoma

## Abstract

**Objectives:**

Langerhans cells (LCs) are epithelial antigen‐presenting cells (APC) contributing to immune surveillance. LCs depend on interleukin 34 (IL34) production by epithelial cells. This study aimed to uncover mechanisms of alteration of IL34 and LC function in squamous cell carcinoma (SCC).

**Methods:**

Cancer cohort data were used to identify associations between SCC and IL34. ATAC‐seq of keratinocytes (KCs) and LCs from a murine model of epithelial hyperplasia, driven by HPV16 E7 oncoprotein (K14E7), was analysed. Transcriptomic data were used to validate findings. RNAscope, RT‐qPCR, ELISA and confocal imaging was used to analyse IL34 expression and LCs in a spatial context.

**Results:**

*IL34* mRNA is downregulated in human SCCs of the head and neck, the cervix, the lung and the oesophagus, and low *IL34* expression is associated with poor survival. We demonstrate that KCs of K14E7 mice have reduced *Il34* gene accessibility, mRNA and protein, as well as broad changes in promotor accessibility associated with cell adhesion and immune responses. Chromatin accessibility was substantially changed in LCs, including increased accessibility of the *Csf1r* gene, and changes in promotors associated with cytoskeleton arrangement and antigen processing and presentation. We discovered altered spatial LC dendrite organisation in hyperproliferative epithelium.

**Conclusion:**

Squamous cell carcinoma of the cervix, head and neck, oesophagus and lung demonstrate downregulation of IL34, which is associated with poor survival, and with alterations in LC spatial organisation and function. These findings suggest that reduced IL34 expression in SCC may contribute to impaired local immunity through LC dysregulation.

## Graphical abstract

IL34 is critical for the development and maintenance of Langerhans cells in squamous epithelium. This study shows that low expression of IL34 determines poor survival in squamous cell carcinomas. We demonstrate that the downregulation of IL34 transcript and protein in hyperproliferative squamous epithelial cells is because of the reduction in chromatin accessibility at the enhancer region of the *Il34* gene. This epigenetic alteration is associated with substantial chromatin changes at genes pivotal to the antigen processing and presentation machinery of Langerhans cells.

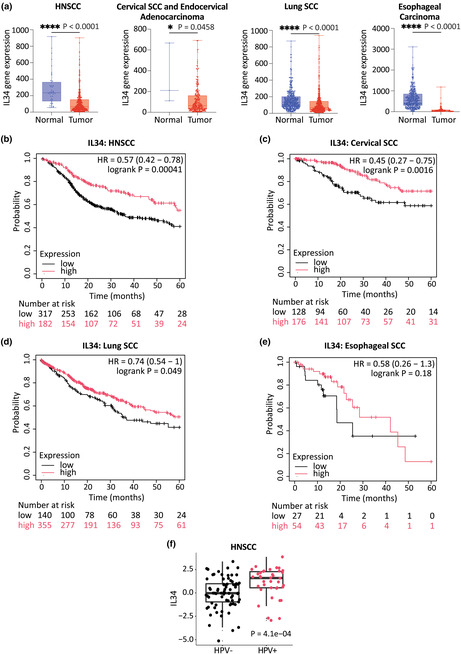

## Introduction

Epithelial hyperplasia is a common response to chronic epithelial inflammation, injury or infection^1^ and may predispose to the development of cutaneous and mucosal squamous cell carcinoma (SCCs).^2^ Hyperplastic epithelium is associated with alterations to epithelial cell gene expression,^3^, ^4^ through transcriptional regulation.^5^ Understanding the molecular basis of epithelial hyperplasia may facilitate the development of targeted therapeutic strategies for epithelial cancer.

A transgenic mouse expressing the E7 oncoprotein of the high‐risk human papillomavirus (HPV) type 16 under the control of the keratin 14 promoter (K14E7) exhibits epithelial hyperplasia^6^ reminiscent of HPV‐infected human epithelium^7^ and of actinic keratosis.^8^ The K14E7 epithelium contains an immune‐suppressive immune infiltrate, including regulatory T cells, mast cells and NKT cells.^9^, ^10^, ^11^, ^12^, ^13^, ^14^ The K14E7 mouse thus enables investigation of immune regulation in epithelial proliferative diseases.^15^ Langerhans cells (LCs) are the only professional antigen‐presenting cells (APCs) in the cutaneous epithelium in steady state.^16^ Our team reported that steady‐state murine LCs exist in different cell states, including phagocytic, proliferative, fully mature stimulatory and semi‐mature inhibitory cell states.^4^ However, in K14E7 hyperproliferative epithelium, fully mature LC cell states are absent, while semi‐mature LCs are enriched.^4^ This aberration in K14E7 LC transcriptional profiles corresponds with reduction in the expression of surface markers, including Langerin (CD207), CD11b, MHCII and EpCAM, and with elevated expression of immune‐modulatory enzymes and cytokines, including *Ido1*, *Arg1* and *Il6*.^17^ These cells exhibit a diminished capacity for antigen uptake both *in vitro* ^17^ and *in vivo*,^18^ as well as in antigen processing, when compared to WT LCs.^19^ Interestingly, culturing K14E7 LCs *ex vivo* restores their capacity to present antigens and prime T cells.^17^, ^19^ This suggests that the hyperplastic epithelium's microenvironment continuously provides signals that maintain LC dysfunction.

Identified as an alternative ligand for the colony‐stimulating factor 1 (CSF1), IL34 has been shown to be essential for the maintenance and development of LCs in mouse skin.^20^, ^21^, ^22^ LC presence in the murine epidermis is strictly dependent on IL34 and CSF1R. In *Il34* knockout mice, LCs are absent^20^, ^22^ and, similarly, they are absent in *Csf1r*‐deficient mice.^23^ However, LCs are present in mice with an inactivating mutation in the *Csf1* gene, indicating that LC presence is CSF1‐independent.^23^ We have previously demonstrated the alteration of keratinocytes (KCs) in K14E7 skin at transcriptomic levels,^3^ and predicted an absence of IL34‐CSF1R ligand receptor interaction.^4^ To deepen our understanding of genetic regulation in epithelial hyperplasia, in this study, we assessed the chromatin accessibility landscape broadly and within the *Il34* and *Csf1r* gene loci in KCs and LCs between normal and hyperplastic epithelium.

## Results

### *Il34* mRNA expression is reduced in SCC, and is associated with poor survival

**Figure 1:**
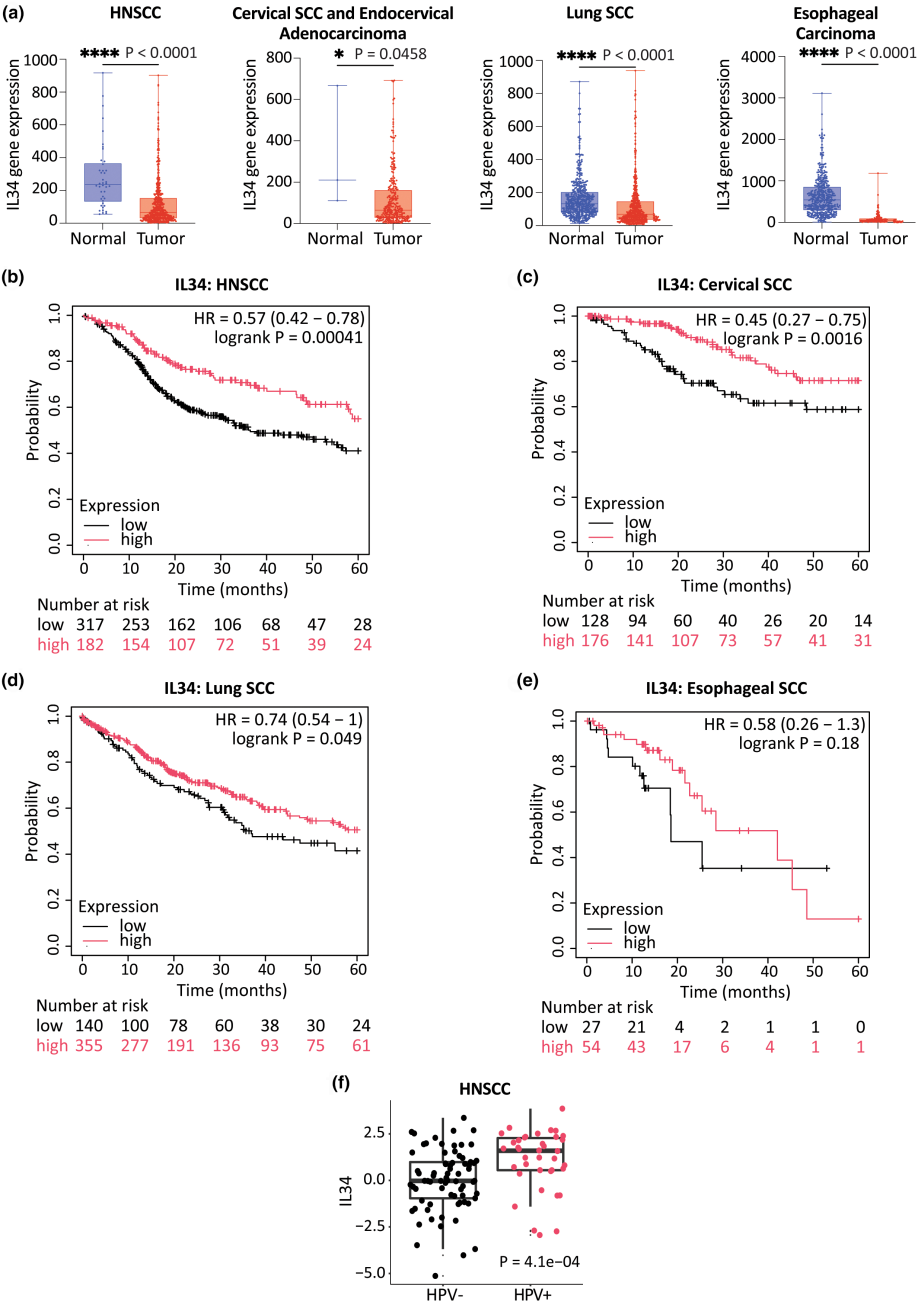
Mucosal SCC and K14E7 KCs express reduced levels of *IL34*. **(a)** TNM data.^50^ Boxplots of *IL34* gene expression in HNSCC (normal = 44, tumor = 500), cervical SCC and endocervical adenocarcinoma (normal = 3, tumor = 304), lung SCC (normal = 476, tumor = 501) and esophageal carcinoma (normal = 418, tumor = 161). Unpaired two‐tailed Mann–Whitney *U*‐test, *P < 0.05; ****P < 0.0001. **(b–e)** Kaplan–Meier^68^ plots based on IL34 expression of HNSCC **(b)**, cervical SCC **(c)**, lung SCC **(d)** and oesophageal SCC **(e)** samples. **(f)** The comparison between *IL34* expression and HPV status in HNSCC.

We previously showed that *IL34* mRNA expression is downregulated in cutaneous and mucosal squamous epithelial hyperplasia, including high‐grade cervical intraepithelial neoplasia (CIN3), psoriasis and eczema.^4^ To expand on these findings, we compared *IL34* mRNA expression in normal and tumor tissues from patients with mucosal SCCs. Our analysis demonstrated a consistent pattern of reduced *IL34* mRNA expression in SCC of the head and neck, cervix, lung and oesophagus compared to normal tissues (Figure 1a). Furthermore, we observed significantly reduced survival in patients exhibiting low *IL34* expression in cervical SCC, in head and neck SCC (HNSCC) and in lung SCC (Figure 1b–d). Noteworthy, a similar survival trend was also observed in patients with oesophageal SCC, although the statistical power in this data set was limited (Figure 1e). In HNSCC, we further observed higher *IL34* levels in HPV+ HNSCC than in HPV‐ HNSCC (Figure 1f), aligning with previous findings of increased immune activity in HPV+ HNSCC.^24^ Overall, these findings are consistent with a role of IL34 in determining outcome of SCC.

### Epithelial hyperplasia is associated with reduced *Il34* expression

**Figure 2:**
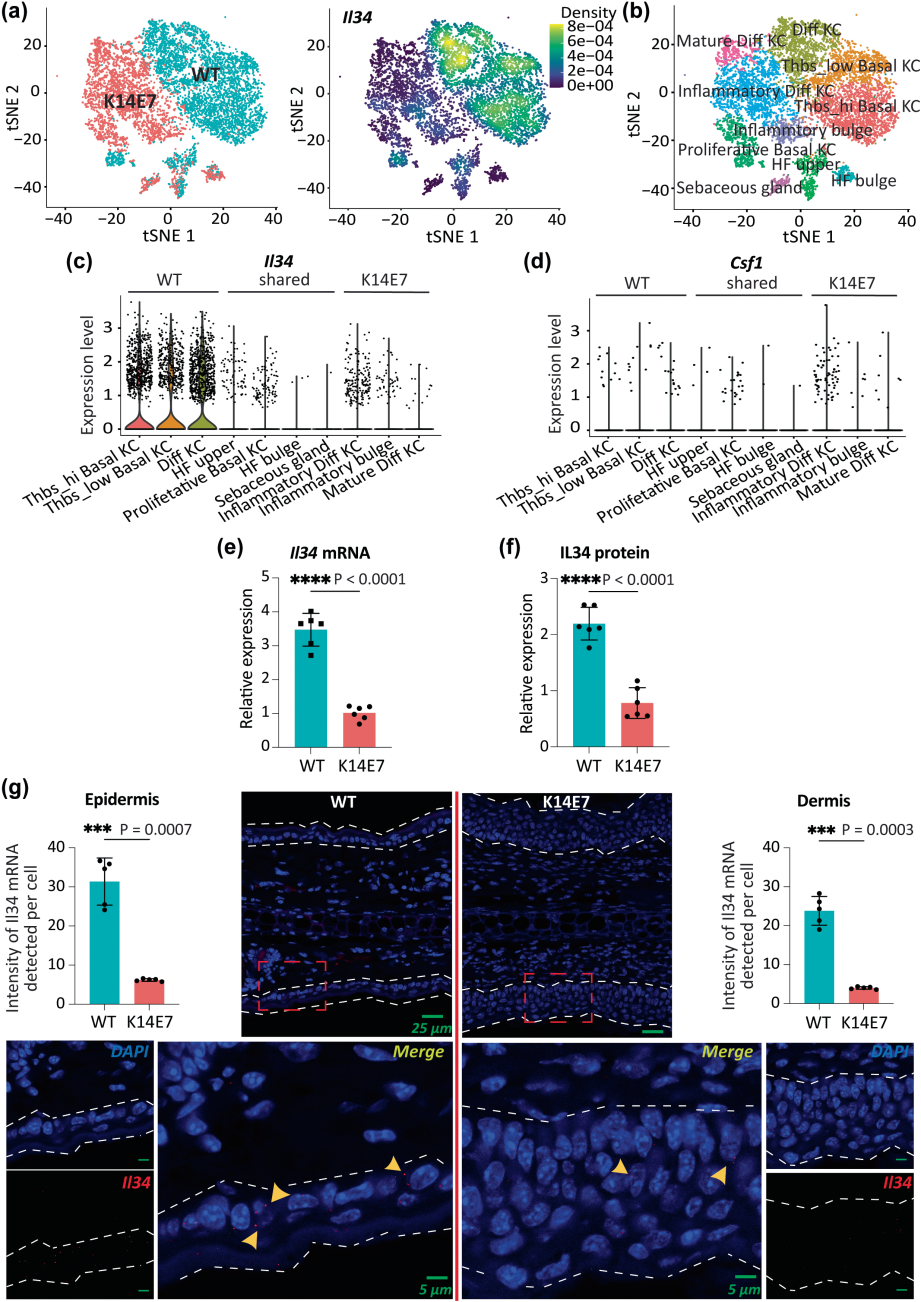
K14E7 KCs express reduced levels of *Il34*. **(a)** scRNA‐seq data^3^ was used to visualise **(a)** sample groups (left) and *Il34* expression (right) using Nebulosa, **(b)** cell states as *t*‐SNE plot, **(c)** *Il34* expression across K14E7 and WT cell states as violin plot and **(d)** *Csf1* expression across K14E7 and WT cell states as violin plot. **(e, f)** Comparison of *Il34* mRNA expression (normalised by *Rps6*, *Rpl5*, *eIF3a*) **(e)** and IL34 protein expression **(f)** between K14E7 and WT epithelial skin cell lysates (*n* = 6 combined from 2 independent experiments). Unpaired *t*‐test with Welch's correction. ****P < 0.0001. **(g)** Representative of *Il34*‐targeted *in situ* hybridisation using cross‐sections of ears. The dashed line indicates the epithelium. Scale bars represent 25 μm for the top images and 5 μm for the bottom images. *Il34* expression levels per cell. Unpaired *t*‐test with Welch's correction. ***P < 0.001. All error bars represent standard deviation (SD).

Employing single‐cell RNA sequencing (scRNA‐seq) data from sorted KCs from a murine K14E7 transgenic model of epithelial hyperplasia,^3^ we identified significantly lower *Il34* mRNA (adjusted *P* = 2.84E−174) in K14E7 KCs than non‐transgenic wild‐type (WT) KCs (Figure 2a). Basal and differentiated WT‐derived KCs primarily produced Il34 transcripts, while proliferative KCs and all K14E7‐derived KC cell states exhibited lower contributions (Figure 2b and c). Only few KCs expressed *Csf1* transcripts, with no significant difference between WT and K14E7 (Figure 2d), suggesting *Csf1* does not compensate for the lack of *Il34* in epithelial hyperplasia. We investigated other macrophage survival factors, such as GM‐CSF (CSF2), FLT3L or TGF‐β, with *Csf2* and *Flt3lg* not detected, and *Tgfb1* expressed at low, non‐differential levels in our existing data, which includes bulk RNA‐seq data^8^ and scRNA‐seq data of epithelial cells from K14E7 and WT mice.^3^ Reduction in *Il34* mRNA was further validated using RT‐qPCR (Figure 2e). Correspondingly, decreased IL34 protein was observed using ELISA (Figure 2f). *In situ* hybridisation revealed higher expression of *Il34* mRNA in the epidermis than in dermis, and reduced *Il34* expression per cell in K14E7‐derived KCs (Figure 2g). Collectively, the results demonstrate that squamous epithelial hyperplasia is associated with decreased expression of epithelial *Il34* mRNA and IL34 protein.

### The chromatin accessibility landscape in hyperproliferative KCs

**Figure 3:**
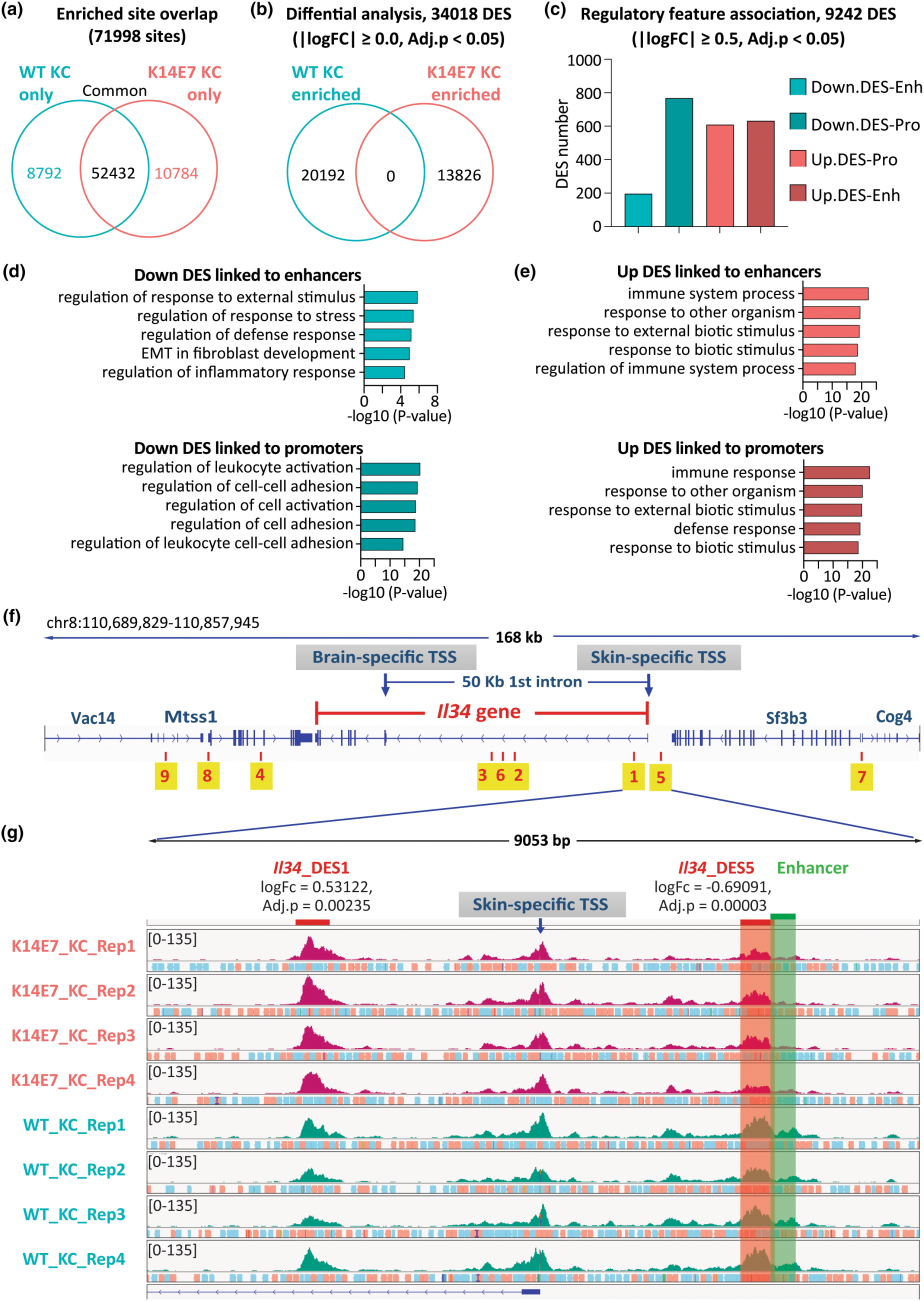
Chromatin accessibility is significantly changed in hyperplastic KCs and reduced at the *Il34* gene locus. **(a, b)** Venn diagrams, generated in DiffBind, show the overlap of the enriched sites identified after defining the consensus peakset **(a)** and after differential enrichment analysis **(b)**. Epithelial samples from four independent experiments, each pooling cells from 6 WT or 3 K14E7 mice. **(c)** The association of DES with enhancers and promoters. **(d, e)** Gene ontology analysis of less accessible DES **(d)** and more accessible DES **(e)** that were associated with enhancer and promoter regions. **(f)** IGV visualisation of ATAC‐seq peaks at the *Il34* gene and DES within its locus spanning 50 kb upstream and downstream. **(g)** IGV visualisation of *Il34*_DES1 (logFC = 0.53122, Adj.*P* = 0.00235) and *Il34*_DES5 (logFC = −0.69091, Adj.*P* = 0.00003). The red bar highlights *Il34*_DES5 overlapping with an upstream enhancer of *Il34* highlighted by the green bar.

The gene expression profile of K14E7 KCs is vastly different from WT KCs.^3^ To investigate whether altered gene expression correlates with changes in chromatin accessibility, we performed bulk ATAC‐seq on sorted KCs from K14E7 and WT epithelium (Supplementary figure 1). After quality assessments, we obtained approximately 50 million reads per sample (Supplementary figure 2a). We established a consensus peak set comprising 71 998 peaks, where 52 432 were common to both WT and K14E7, 8782 were unique to WT, and 10 784 were exclusive to K14E7 (Figure 3a). Differential analysis revealed 34 018 differentially enriched sites (DES) between WT and K14E7 KCs, with 20 192 displaying reduced accessibility and 13 826 showing greater accessibility in K14E7 KCs (Figure 3b). To increase biological significance, we applied an absolute logFC cut‐off of 0.5, resulting in a reduction to 9242 DES. Approximately 50% of DES overlapped with gene bodies, while 40% were in intergenic regions (Supplementary figure 2b). The top five more accessible DES in K14E7 KCs were associated with *Nppb*, *Kirrel3*, *Kcna4*, *Lrrc38* and *Plpp4* gene loci, while the top five less accessible DES were linked to *Kif6*, *4930452A19Rik*, *Kcnt2*, *Kcnip4* and *C6* gene loci (Supplementary figure 2c). Analysing the regulatory feature association revealed that K14E7 KCs had 767 DES with decreased promoter access and 631 DES with increased promoter access, and 195 DES with decreased enhancer access and 608 with increased enhancer access (Figure 3c). Gene ontology analysis suggested that less accessible enhancer and promoter regions were linked to immune regulation and cell adhesion processes (Figure 3d), while more accessible regions were associated with immune responses to biotic stimuli and antigen presentation (Figure 3e). Overall, these findings indicate significant changes in chromatin accessibility in KCs from epithelial hyperplasia with extensive alterations in access to promoter and enhancer regions, suggesting alterations in cell‐to‐cell organisation and an activated immune response.

### The *Il34* gene locus in hyperproliferative KCs is less accessible

The *Il34* gene in mice has two transcription start sites (TSS) that are mainly active in the brain and skin (FANTOM 5 data^25^). The brain‐specific TSS is at the first coding exon, while the skin‐specific TSS is further upstream at the first non‐coding exon, separated by a large 50 kb intron (Figure 3f). Conducting a DES analysis at the *Il34* gene locus spanning 50 kb upstream and downstream, we identified nine DES, with seven significantly less accessible in K14E7 KCs compared to WT KCs (Table 1 and Figure 3f). From the distribution of these sites, we identified that *Il34*_DES1 and *Il34*_DES5 exhibited close proximity to the skin‐specific TSS. Notably, *Il34*_DES5 partially overlapped with the upstream enhancer of the *Il34* gene (Figure 3g), implying perturbations in regulatory elements governing *Il34* gene expression.

**Table 1:** DES at *Il34* gene locus in KCs extended by 50 kb upstream to 50 kb downstream

DES	Coordinates	Width	LogFC	*P*‐value	Adj.*P*
*Il34*_DES1^a^	chr8:110 803 073–110 803 473	401	0.53122	0.00070	0.00235
*Il34*_DES2	chr8:110 780 123–110 780 523	401	0.40227	0.02109	0.04483
*Il34*_DES3	chr8:110 775 709–110 776 109	401	−0.81453	0.00272	0.00770
*Il34*_DES4	chr8:110 731 362–110 731 762	401	−0.77457	0.00003	0.00016
*Il34*_DES5^b^	chr8:110 808 291–110 808 691	401	−0.69091	0.00001	0.00003
*Il34*_DES6	chr8:110 777 822–110 778 222	401	−0.64581	0.00011	0.00048
*Il34*_DES7	chr8:110 846 865–110 847 265	401	−0.61925	0.00003	0.00016
*Il34*_DES8	chr8:110 721 241–110 721 641	401	−0.55986	0.00006	0.00026
*Il34*_DES9	chr8:110 713 090–110 713 490	401	−0.38404	0.00766	0.01880

Positive logFC means increased chromatin accessibility in K14E7 KCs, while negative logFC indicates decreased chromatin accessibility in K14E7 KCs.

^a^ Proximity to the skin‐specific *Il34* TSS.

^b^ Proximity to the skin‐specific *Il34* TSS and overlap with *Il34* upstream enhancer.

### The LC chromatin accessibility landscape

**Figure 4:**
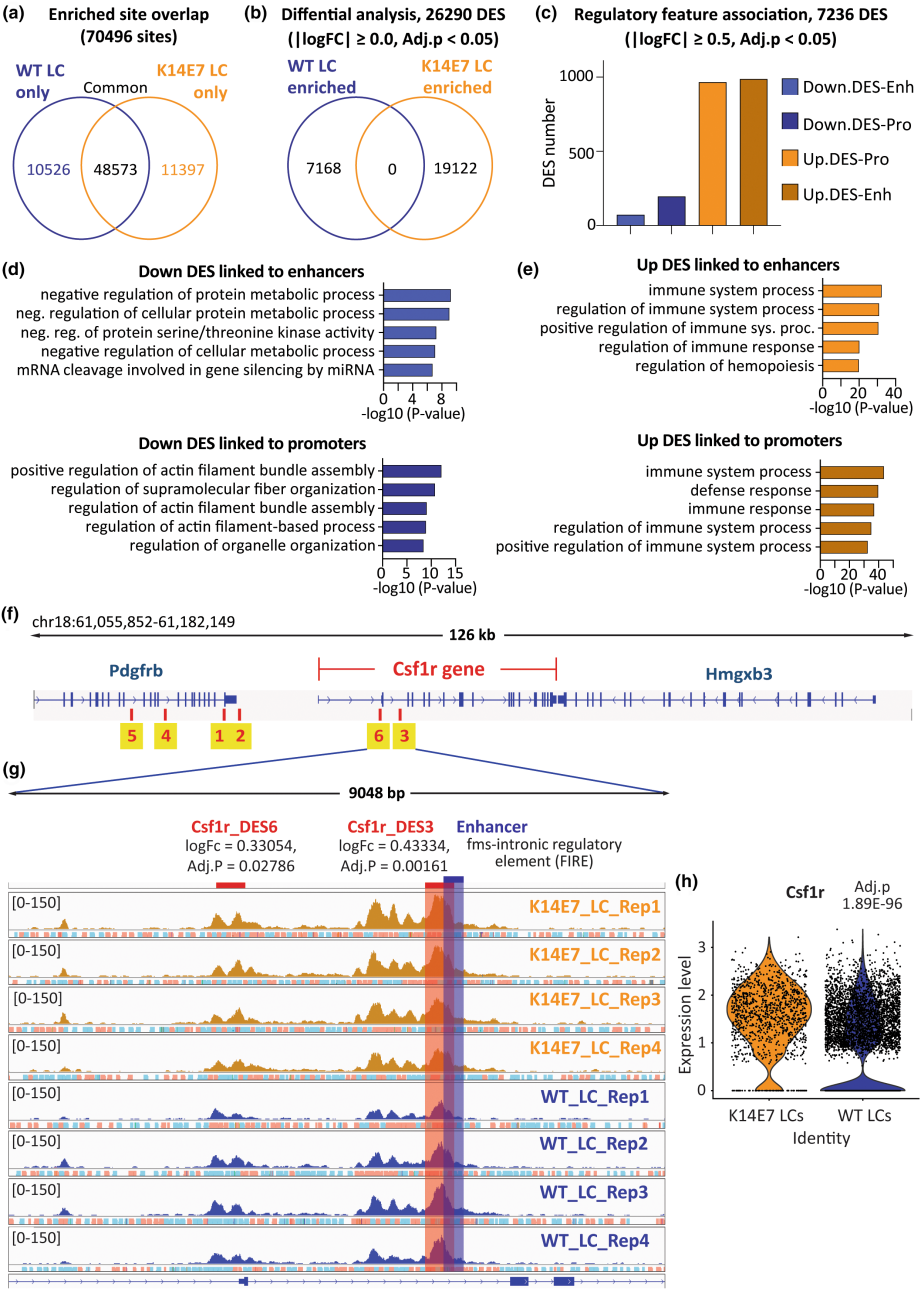
Chromatin accessibility is significantly changed in LCs within hyperplastic epithelium and enhanced at *Csf1r* locus. **(a, b)** Venn diagrams, generated in DiffBind, show the overlap of the enriched sites identified after defining the consensus peakset **(a)** and after differential enrichment analysis **(b)**. Sorted LC samples from four independent experiments, each pooling cells from six WT or three K14E7 mice. **(c)** The association of DES with enhancers and promoters. **(d, e)** Gene ontology analysis of less accessible DES **(d)** and more accessible DES **(e)** that were associated with enhancer and promoter regions. **(f)** IGV visualisation of *Csf1r* gene and DES within its locus spanning 50 kb upstream and downstream. **(g)** IGV visualisation of *Csf1r*_DES3 (logFC = 0.43334, Adj.*P* = 0.001611) and *Csf1r*_DES6 (logFC = 0.33054, Adj.*P* = 0.02786). The red bar highlights *Csf1r*_DES3 overlapping with FIRE enhancer highlighted by the blue bar. **(h)** scRNA‐seq data^4^ was used to analyse the expression of *Csf1r* gene in K14E7 and WT LCs presented as a violin plot.

As introduced, we previously established the transcriptional, phenotypic and functional alterations of K14E7 LCs.^4^, ^17^, ^18^, ^19^ To investigate whether chromatin accessibility changes are associated with these alterations, we conducted bulk ATAC‐seq on sorted K14E7 and WT LCs (Supplementary figure 1). Post‐alignment and quality control, each sample yielded approximately 50 million reads (Supplementary figure 2a). We established a consensus peak set comprising 70 496 peaks, where 48 573 were common to both WT and K14E7, 10 526 were unique to WT, and 11 397 were exclusive to K14E7 (Figure 4a). Differential analysis revealed 26 290 DES between WT and K14E7 LCs, with 7168 displaying reduced accessibility and 19 122 showing greater accessibility in K14E7 LCs (Figure 4b). To focus on changes more likely to have biological consequences, we applied an absolute logFC cut‐off of 0.5, resulting in 7236 DES. Approximately 50% of DES intersected with gene body segments, 37.44% were in distal intergenic regions, and 11% with promoter regions (Supplementary figure 2d). The top five more accessible DES in K14E7 LCs were associated with *Olfr810, Slc13a1, Bcl10, Olfr808* and *Cmtm6* gene loci, while the top five less accessible DES were linked to *Tnfaip2*, *Cd83*, *Zbtb10*, *Mir592* and *Iqcn* gene loci (Supplementary figure 2e). Analysing the distribution of these DES at regulatory regions, we observed a large number of K14E7‐derived LC‐enriched DES overlapped with enhancer and promoter regions (Figure 4c). Gene ontology analysis revealed that less accessible enhancer regions were dominantly involved with metabolic processes, while less accessible promoter regions were involved in cell adhesion, organisation of actin filaments, cytoskeleton structures and stress fibre assembly (Figure 4d). Conversely, more accessible enhancer and promoter regions in K14E7 LCs were associated with immune responses (Figure 4e). Overall, these findings demonstrate a proportionally increased chromatin accessibility of LCs in hyperproliferative epithelium, particularly notable in the pronounced enrichment at promoter and enhancer regions.

### LCs resident to epithelial hyperplasia have increased chromatin accessibility and transcript of the *Csf1r* gene

We determined whether epithelial hyperplasia was associated with chromatin changes in the *Csf1r* gene locus. We identified six DES within the *Csf1r* locus spanning 50 kb upstream and downstream with all sites demonstrating increased accessibility in K14E7 LCs compared to WT (Table 2 and Figure 4f). Notably, the *Csf1r*_DES3 partially overlapped with a known *Csf1r* enhancer (Figure 4g), the *fms*‐intronic regulatory element (FIRE), which is critical for *Csf1r* transcription, and its deletion is associated with the loss of LCs.^26^ *Csf1r*_DES6 overlapped with a macrophage‐specific TSS and the upstream promoter element (Figure 4g). Our scRNA‐seq data revealed a significant increase in *Csf1r* transcripts (adjusted *P* = 1.89E−96) in K14E7 LCs (Figure 4h). These findings demonstrate that the *Csf1r* gene in K14E7 LCs exhibits enhanced accessibility for transcription, resulting in increased *Csf1r* gene expression.

**Table 2:** DES at *Csf1r* locus in LCs extended by 50 kb upstream to 50 kb downstream

DES	Coordinates	Width	LogFC	*P*‐value	Adj.*P*
*Csf1r*_DES1	chr18:61 083 100–61 083 500	401	1.18853	1.569E−08	1.935E−07
*Csf1r*_DES2	chr18:61 085 308–61 085 708	401	1.21678	2.320E−07	2.284E−06
*Csf1r*_DES3^a^	chr18:61 108 443–61 108 843	401	0.43334	3.490E−04	1.611E−03
*Csf1r*_DES4	chr18:61 074 606–61 075 006	401	0.66097	5.574E−04	2.431E−03
*Csf1r*_DES5	chr18:61 069 779–61 070 179	401	0.35667	5.969E−03	1.885E−02
*Csf1r*_DES6^b^	chr18:61 105 563–61 105 963	401	0.33054	9.449E−03	2.786E−02

Positive logFC means increased chromatin accessibility in K14E7.

^a^ Overlap with FIRE enhancer.

^b^ Overlap with promotor region.

### Spatial organisation of CSF1R‐expressing cells and of their dendrites in epithelial hyperplasia is disturbed

**Figure 5:**
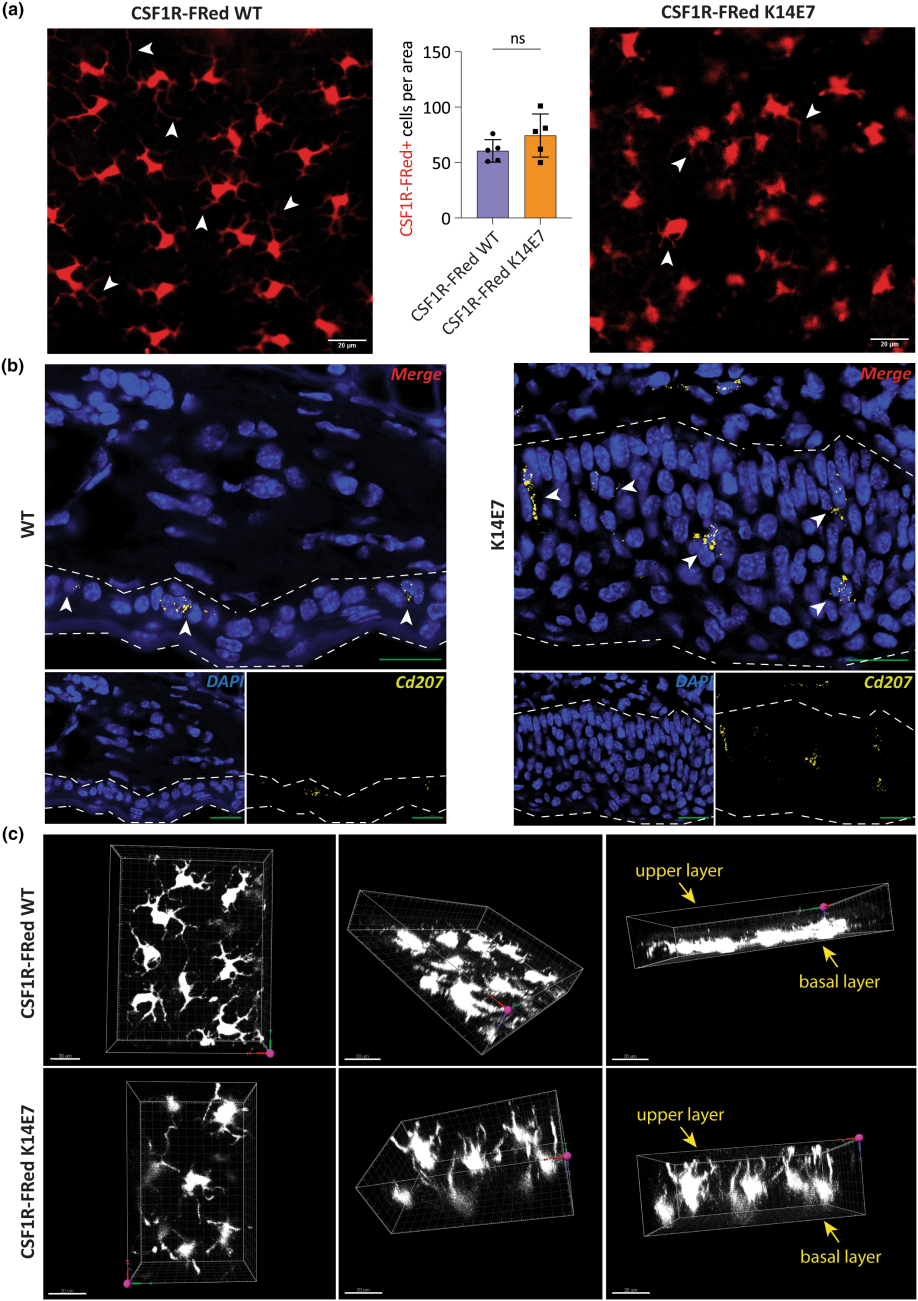
Spatial organisation of CSF1R‐expressing cells and of their dendrites in the hyperproliferative epithelium. **(a)** Confocal imaging of CSF1R‐Fred K14E7 and CSF1R‐Fred WT skin. Arrowheads show positions of dendrites. Scale bars represent 20 μm. Epithelial CSF1R‐Fred+ cells were enumerated following confocal imaging; unpaired *t*‐test with Welch's correction; ns, nonsignificant; error bars represent SD. **(b)** Representative target *Cd207* RNA expression at a single‐cell level using RNAscope on fresh frozen ear pinnae of K14E7 and WT mice. The dashed line indicates the epithelium. Arrowheads show positions of LCs. Scale bars represent 20 μm. **(c)** 3D images of CSF1R‐Fred K14E7 and CSF1R‐Fred WT skin. The xy surface (indicated by red and green arrows, respectively) indicates the outermost layers of the skin. Scale bars represent 20 μm.

Promotor accessibility analysis suggested that K14E7 KCs had decreased promotor activity in cell adhesion, while K14E7 LCs showed decreased promotor activity in cytoskeleton organisation and actin filament assembly. This may alter the spatial LC organisation and dendrite formation in hyperproliferative epithelium. To study LC distribution and phenotype, we crossed K14E7 animals with CSF1R‐FRed reporter mice.^27^ In whole mount images, CSF1R‐FRed+ cell numbers per area were similar in K14E7 and WT epithelium, but K14E7 CSF1R‐FRed+ cells appeared to have fewer horizontally oriented dendrites from a top view of the skin; however, an in‐focus image of LCs from the hyperplastic K14E7 epithelium was difficult to obtain (Figure 5a). Using cross‐sections and *Cd207*‐targeted *in situ* hybridisation, we observed that WT LCs were primarily situated in the basal layer of the epidermis, while K14E7 LCs were distributed throughout the hyperplastic epithelium, with mostly vertically oriented dendrites compared to the horizontal dendrites of WT LCs (Figure 5b). This was confirmed with 3D imaging of CSF1R‐FRed+ cells in K14E7 and WT epithelium (Figure 5c, Supplementary videos 1 and 2), indicating that LC dendrite formation is not impaired but is directed throughout the additional epithelial hyperplastic layers.

### The chromatin landscape of the antigen processing and presentation machinery is dysregulated in LCs within hyperplastic epithelium

**Figure 6:**
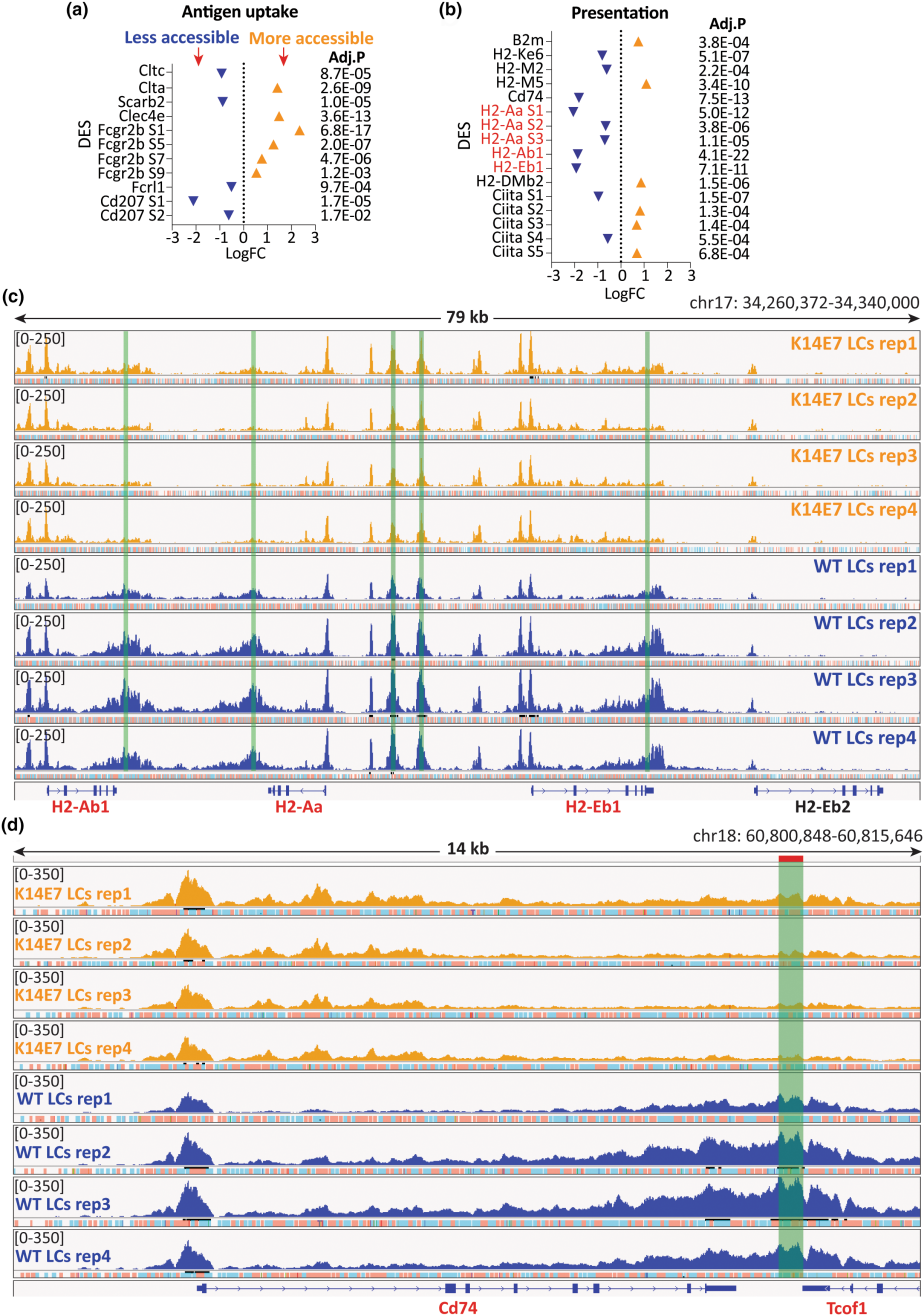
Chromatin accessibility is significantly decreased at genes involved in antigen presentation in K14E7 LCs. **(a, b)** DES of genes involved in the antigen uptake **(a)** and the antigen presentation pathway **(b)** were plotted based on logFC, with corresponding adjusted *P*‐values provided on the right. Negative logFC values indicate reduced accessibility in K14E7 LCs, and positive values signify increased accessibility. **(c, d)** IGV visualisation of ATAC‐seq peaks at classical MHCII gene loci **(c)** and *Cd74* **(d)**. Green bars indicate identified DES.

**Figure 7:**
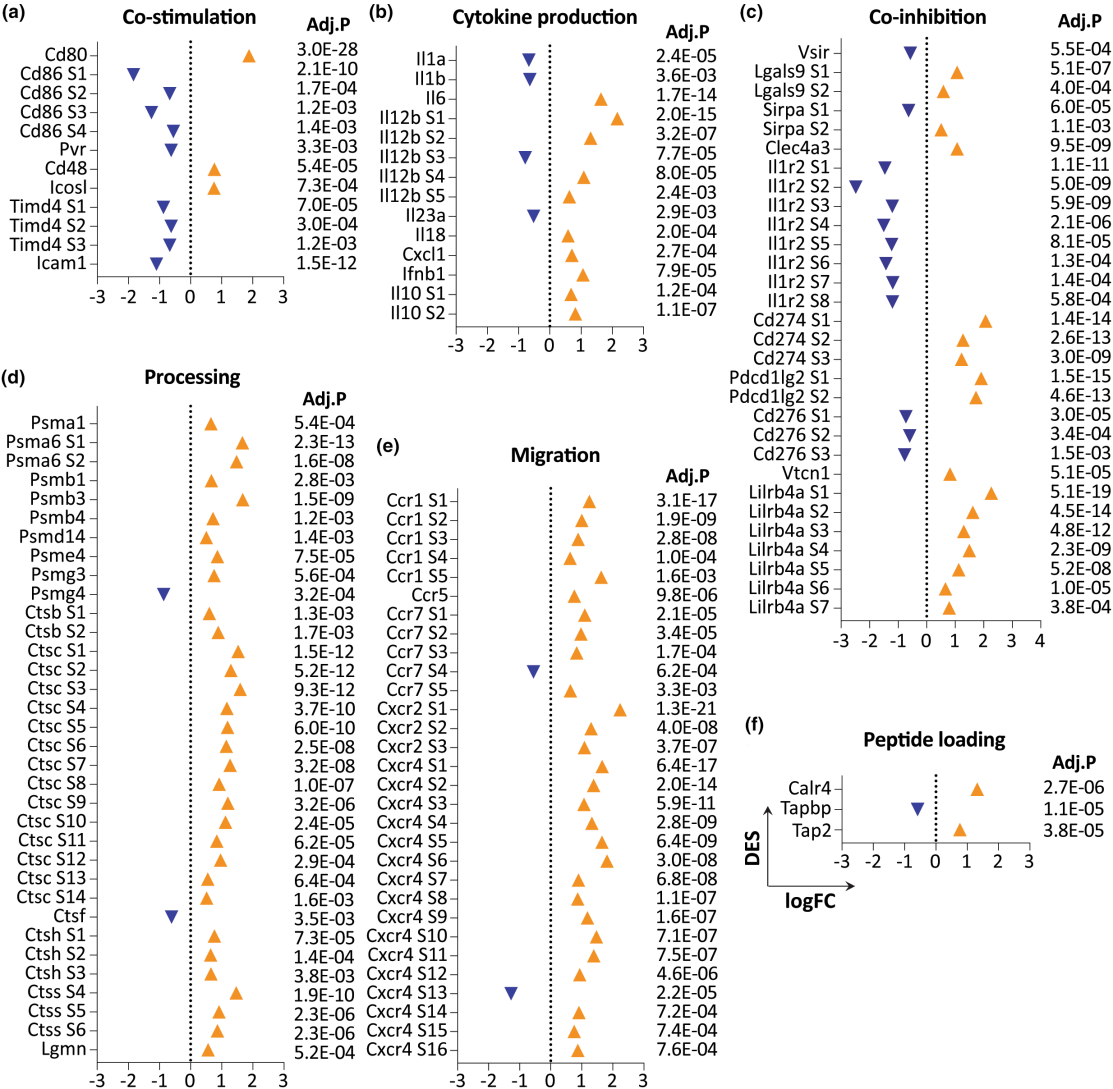
Chromatin accessibility alterations at genes involved in the antigen processing and presentation machinery. IGV visualisation of ATAC‐seq peaks at genes involved in co‐stimulation **(a)**, cytokine production **(b)**, co‐inhibition **(c)**, processing **(d)**, migration **(e)** and peptide loading **(f)**.

The impairment of K14E7 LCs in antigen uptake, processing and presentation described previously^17^, ^18^, ^19^ prompted us to investigate chromatin accessibility changes in genes involved in the antigen processing and presentation machinery (APPM). We established a comprehensive APPM gene list based on three existing sources (Supplementary table 1).^4^, ^28^, ^29^ In antigen uptake, there were diverse changes with decreased accessibility at key genes involved in phagocytosis, including clathrin (*Clt*), scavenger receptor (*Scarb2*), Fc Receptor‐Like Protein 1 (*Fcrl1*) and Langerin (*Cd207*) (Figure 6a). Both increased and decreased accessibility were observed at MHCI‐related genes and non‐classical MHCII genes (Figure 6b). Intriguingly, substantially decreased accessibility was identified at classical MHCII genes, including *H2‐Aa*, *H2‐Ab1*, and *H2‐Eb1* and *Cd74* (Figure 6b–d). Notable numbers of co‐stimulatory genes (*Cd86*, *Pvr*, *Timd4*, *Icam1*), as well as pro‐inflammatory genes (*Il1*a, *Il1b*, *Il23a*), exhibited reduced accessibility (Figure 7a and b). Conversely, the anti‐inflammatory gene *Il10* and a number of co‐inhibition genes, including *Cd247* (PD‐L1) and *Pdcd1lg2* (PD‐L2), showed increased accessibility, suggesting a more tolerogenic phenotype (Figure 7b and c). Interestingly, substantially increased accessibility was observed at genes involved in antigen processing and migration (Figure 7d and e), correlating with elevated mRNA levels (Supplementary table 1). Diverse changes were also observed at some peptide‐loading genes (Figure 7f).

Collectively, these data indicate that APPM function is dysregulated in LCs of hyperproliferative epithelium and that the previously observed dysfunction in antigen processing and presentation originates at a dysregulation at chromatin level, and is consistent with alterations in gene expression.

## Discussion

Here, we show that reduced *IL34* gene expression is a hallmark of multiple SCC, including cervical, head and neck, lung and oesophageal SCC, and is linked to poor survival. In a mouse model of epithelial hyperplasia, we show that reduced IL34 expression correlated with alterations in chromatin accessibility at the *Il34* locus, and loss of biological processes referring to cell adhesion. This was further associated with substantial chromatin changes in LCs, affecting morphology and antigen processing and presentation.

IL34 is abundantly expressed in skin and brain tissues,^30^ playing pivotal roles in LC and microglia development,^20^, ^22^ as well as in cellular processes including proliferation, differentiation, migration and cytokine/chemokine production.^31^ However, its involvement in diseases ranges widely from inflammation and autoimmunity to cancer,^32^ with varying impacts depending on the disease context. Elevated IL34 levels have been observed in several solid cancers, including breast,^33^ colorectal,^34^ lung,^35^, ^36^ ovarian^37^ and hepatocellular carcinoma,^38^ often correlating with poor prognosis and tumor progression.^38^, ^39^, ^40^ Contrary to these findings, our study showed a consistent pattern of decreased *IL34* expression in mucosal SCC such as cervical, head and neck, lung and oesophageal SCC, suggesting that low IL34 levels are a common characteristic in these conditions. This is aligned with our previous observations with reduced IL34 levels in epithelial hyperplasia conditions such as CIN3, psoriasis and eczema.^4^ Furthermore, in a spatial context, we have recently described that IL34‐CSF1R co‐expression is specifically absent from the neoplastic region in CIN3 samples, while the adjacent normoplastic tissue regions present normal IL34‐CSF1R co‐expression.^41^ Importantly, we here highlight the clinical significance of low *IL34* expression, as it correlates with poor survival in cervical cancer patients,^4^ in HNSCC and in lung SCC. Availability of large‐scale gene expression and clinical data from other SCC patient cohorts including cutaneous, esophaegeal, anal, vulval, penile and vaginal SCCs will in future enable to establish whether IL34 plays a similar role across these different disease entities.

Human papillomavirus is widely recognised as the primary cause of cervical SCC, and between 38% and 80% of HNSCC cases test positive for HPV.^42^, ^43^, ^44^ Here, we showed that HPV+ HNSCC exhibits higher *IL34* expression levels than HPV− HNSCC. Our recent work revealed that HPV+ HNSCC exhibits a heightened immune activity score,^24^ and research has consistently shown that HPV+ HNSCC typically features a ‘hot’ tumor immune microenvironment, resulting in improved treatment responses and clinical outcomes.^44^, ^45^, ^46^ Collectively, this suggests that IL34 may play a beneficial role in mucosal squamous epithelial cancers, and skin lesions associated with epithelial hyperplasia.

We utilised the K14E7 transgenic mouse model, which demonstrates epithelial hyperplasia resembling a thickened epithelium and gene expression similar to HPV‐driven human neoplastic tissue,^7^ to investigate the genetic mechanisms underlying IL34 downregulation in hyperplastic epithelium. Our study uncovered a corresponding decrease in *Il34* mRNA levels and protein expression. ATAC‐seq analysis of KCs revealed significantly reduced chromatin accessibility at multiple sites within the *Il34* locus, including regions near the skin‐specific TSS and overlap with an upstream enhancer of *Il34*, indicating reduced transcriptional accessibility of *Il34* in hyperplastic epithelium. Although reduction in *Il34* chromatin accessibility was notable in K14E7 KCs, they were less pronounced compared with the reductions observed in mRNA and protein expression, suggesting that factors beyond chromatin accessibility may contribute to suppressing IL34 expression in hyperproliferative epithelium. We explored whether other macrophage survival factors and growth factors such as CSF2, FLT3L, TGF‐β may compensate for the lack of IL34, but gene transcripts were either not present or not differentially expressed in hyperproliferative epithelium. Intriguingly, we observed significantly increased chromatin accessibility at the *Csf1r* locus in LCs within hyperplastic epithelium, correlating with elevated transcript levels. CSF1R, the primary receptor of IL34, is expressed exclusively on mononuclear phagocyte cells,^47^ including LCs. In response to constant ligand stimuli, the receptor is typically downregulated to prevent excessive cellular reactions to the ligand.^48^ This implies that LCs in hyperplastic epithelium may have an increased CSF1R expression because of the reduced availability of IL34, hence not triggering ligand‐mediated downregulation of the receptor. Interestingly though, while genetic depletion of IL34 leads to a complete absence of LCs,^20^, ^22^ reduced IL34 in hyperplastic epithelium did not reduce LC numbers, suggesting that either sufficient IL34 remains in the skin to retain LCs or other factors contribute to LC survival in this environment.

Previously, we demonstrated reduced antigen uptake^17^, ^18^ and impaired antigen processing capability in LCs within hyperplastic epithelium,^19^ associated with decreased expression of classical MHCII genes including *H2‐Aa*, *H2‐Ab1*, *H2‐Eb1* and *Cd74*.^4^ In line with this, we observed reduced chromatin accessibility at classical MHCII and critical phagocytosis genes. Importantly though, we observed both increased and decreased chromatin accessibilities across different antigen processing and presentation pathways, suggesting that LCs resident to epithelial hyperplasia are not simply shut down in the overall antigen processing and presentation machinery, but dysregulated in a complex way. Additionally, consistent with our previous findings, K14E7 LCs exhibited reduced horizontal dendritic extension and a rounded shape from a top view of the skin, but we discovered that dendrite formation was not impaired, but rather oriented vertically through the additional epithelial cell layers.^17^

In conclusion, our research highlights that IL34 downregulation is a common occurrence in diseases associated with epithelial dysplasia, including inflammatory skin diseases and various SCCs such as lung, head and neck, cervical and oesophageal SCC. This aligns with our previous findings of reduced *IL34* transcripts in CIN3, psoriasis and eczema. Moreover, decreased *IL34* levels correlate with poorer survival rates in patients with cervical, lung and head and neck SCC, underscoring its clinical significance. This study provides a comprehensive molecular portrait of the impact of epithelial hyperplasia on LCs, highlighting significant shifts in antigen processing and presentation pathways. These observations contribute to understanding the complex immune dysregulation within the microenvironment of hyperplastic epithelium. Future research is required to provide a cause‐and‐effect link between reduces IL34 production and dysregulated LC function, and whether this signalling axis can be therapeutically manipulated to increase APC function and T cell priming in SCC.

## Methods

### Mice

Eight‐ to twelve‐week‐old WT, K14E7,^49^ CSF1R‐Fred/WT^27^ and CSF1R‐Fred/K14E7 transgenic female mice were used for all experiments. Used littermates were bred either between K14E7 male mice with C57BL/6 females or between CSF1R‐Fred/WT mice with K14E7 mice. All animal experiments were approved by the University of Queensland Animal Ethics Committee (2021/AE000460 and 2023/AE000617). They were maintained under specific pathogen‐free conditions at the Translational Research Institute's Biological Research Facility.

### RNA expression of *IL34* in human tumors

Gene expression of *IL34* in tumor and normal tissues of each cancer set was obtained from the TNMplot database.^50^ Plots were generated using Graphpad Prism 10.

### Kaplan–Meier survival analysis

Survival plots were generated using the Kaplan–Meier Plotter^51^ (kmplot.com) and the following settings: survival (OS), auto selection for best cut‐off (all), follow‐up threshold (60 months). ‘All’ was selected for all other setting options.

### HPV status vs *Il34* expression

Gene expression data from 466 primary tumors of the HNSCC cohort were sourced from Xenabrowser.^52^ The relationship between IL34 expression and HPV status was examined in 111 subjects where HPV status was available. The *P*‐value was calculated using the Wilcoxon rank‐sum test because of non‐normal data distribution.

### scRNA‐seq analysis

Previously acquired scRNA‐seq data of sorted KCs and LCs from WT and K14E7 mice were used.^3^, ^4^ Cell clusters were manually reannotated based on genetic markers. Differential gene expression analysis was performed using the Seurat package^53^ with significant genes (adjusted *P* < 0.001) reviewed for their differentiation cell states and biological relevance. tSNE and gene expression plots were generated using Seurat.

### Preparation of epithelial single‐cell suspensions

Mice were euthanised by CO_2_ asphyxiation, and ear pinnae were harvested and split into dorsal and ventral halves. These halves were digested in PBS with Dispase II (Cat. #04942078001, Roche, Basel, Switzerland) at 37°C for 1 h, and the epidermis was separated from the dermis. The epidermis was chopped into small pieces, further digested with collagenase D (Cat. #11088858001, Roche, Basel, Switzerland) and DNase (Cat. #11284932001, Roche, Basel, Switzerland) in PBS at 37°C for 30 min and filtered through a 70‐μm cell strainer. The resulting single‐cell suspension was used for RNA and protein extraction, sorting and flow cytometry.

### RT‐qPCR

RNA from epithelial single‐cell suspensions was extracted using the RNeasy Mini kit (Cat. #74104, QIAGEN, Germantown, USA) and DNase‐treated with the TURBO DNA‐free™ Kit (Cat. #AM1907, Thermo Fisher Scientific, Waltham, USA). RNA was reverse‐transcribed into cDNA using Superscript III RT (Cat. #18080093, Thermo Fisher Scientific, Waltham, USA) with the following conditions: 25°C for 5 min, 50°C for 90 min and 70°C for 15 min. Quantitative PCR (qPCR) was performed using the QuantStudio™ 7 Flex Real‐Time PCR System (Thermo Fisher Scientific, Waltham, USA) with specific primers (Supplementary table 2).

### ELISA

IL34 expression in the epithelium was measured from total protein from lysed epithelial cells using the LEGEND MAX™ Mouse IL34 ELISA Kit (Cat. #439107, BioLegend, San Diego, USA) following the manufacturer's instructions. Absorbances were measured using a Multiskan GO (Thermo Fisher Scientific, Waltham, USA).

### RNA *in situ* hybridisation

OCT‐embedded fresh frozen skin tissues were sectioned at 10 μm thickness. Target probes (Cat. #ADV1222201T3 and #ADV452521T2, Advanced Cell Diagnostics, Newark, USA) were used with the RNAscope™ HiPlex12 Reagent Kit v2 Standard Assay Manual. Slides were fixed in 4% paraformaldehyde for 1 h at room temperature, washed with PBS and dehydrated with ethanol (50%, 70% and 100% for 5 min each). Protease IV (Cat. #322340, Advanced Cell Diagnostics, Newark, USA) was applied for 30 min at room temperature, followed by incubation with the probe mix at 40°C for 2 h. Probes were amplified and fluorescently labelled using the Detection‐RNAscope HiPlex 12 Reagents Kit (Cat. #324409, Advanced Cell Diagnostics, Newark, USA). Slides were washed, stained with DAPI and mounted with ProLong Gold Antifade Mountant (Cat. #P36930, Thermo Fisher Scientific, Waltham, USA). Imaging was performed on the LSM900 Airyscan confocal microscope, and images were stitched and adjusted using the ZEN software (version 3.2). Signals from different rounds were merged using the HiPlex v2—Image Registration Software. The cell segmentation was performed using the StarDist^54^ in QuPath^55^ and the average pixel intensity of each targeted mRNA probe signal per cell was quantified.

### Cell sorting

Epithelial single‐cell suspensions were stained with 300 μL of anti‐mouse CD16/CD32 antibody at a 1/100 dilution (Cat. #553141, BD Biosciences, New Jersey, USA) and Fixable Aqua Dead Cell Stain at a 1/200 dilution (Cat. #L34965, Thermo Fisher Scientific, Waltham, USA) for 10 min on ice in the dark. Samples were washed with PBS and further incubated for 20 min with 300 μL antibody cocktail including anti‐mouse CD45‐PerCP‐Cy5.5 at a 1/400 dilution (Cat. #103131, Biolegend, San Diego, USA), EpCAM‐PE at a 1/400 dilution (Cat. 118205, Biolegend, San Diego, USA) and MHCII‐APC‐Cy7 at a 1/400 dilution (Cat. #107627, Biolegend, San Diego, USA). Samples were washed with PBS and resuspended in PBS with 2% FBS and 5 mM EDTA. For sorting, live CD45^−^ cells (KCs) and live CD45^+^ MHCII^+^ EpCam^+^ cells (LCs) were sorted into 100% FBS using the MoFlo Astrios Cell Sorter (Beckman Coulter, Brea, USA).

### ATAC‐seq library preparation and sequencing

Bulk ATAC‐seq was performed on 16 samples, comprising four cell types: K14E7_KC, WT_KC, K14E7_LC and WT_LC, sorted from four independent experiments. Each experiment used pools of epidermal single‐cell suspensions from six WT or three K14E7 mice. 50 000 LCs and 50 000 KCs from WT and K14E7 mice were processed for ATAC‐seq. Cell pellets were washed with ice‐cold PBS, lysed in cold lysis buffer (1 M Tris–HCl pH 7.4, 5 M NaCl, 1 M MgCl_2_, 10% NP‐40, 10% Tween‐20, nuclease‐free water), centrifuged and washed again. Nuclei were resuspended in transposition buffer (25 μL Tagment DNA (Cat #FC‐121‐1030, Illumina, San Diego, USA), 2.5 μL Tn5 Transposase (Cat #FC‐121‐1030, Illumina, San Diego, USA), 22.5 μL nuclease‐free water) and fragmented at 37°C for 30 min. Fragmented chromatin was purified and amplified using PCR, then further purified and eluted in nuclease‐free water. Library quality was assessed using the High Sensitivity D1000 ScreenTape Assay, with successful libraries having quantities above 10 ng μL^‐1^. SPRI select was performed to exclude fragments outside the 100 bp to 1 kb range. Pair‐end sequencing was done using NGS, aiming for 50+ base pairs per read and approximately 50 million reads per sample.

### ATAC‐seq analysis

Raw sequences were qualified using FastQC.^56^ Low‐quality and adapter bases were removed using fastp^57^ and mapped to the mouse genome GRCm38 (mm10) using Bowtie2.^58^ BAM files were generated by using SAMtools^59^ and Picard.^60^ Peaksets were derived by using MACS3 peak caller.^61^ Consensus peaksets were established by restricting analysis to peaks identified consistently in all replicates. Differential analysis was executed by default using DESeq2 (ref. 59) interrogated in DiffBind package.^62^ BEDtools^63^ was used to define the association of DES with known mouse promoters and enhancers from the Fantom database.^25^, ^64^, ^65^ Annotation of DES was performed using ChIPseeker package.^66^ Genomic Regions Enrichment of Annotations Tool (GREAT)^67^ was used to identify biological processes.

### Confocal microscopy

Direct imaging of whole ears was conducted with minimal disturbance through the ear surface. Images were captured using the Olympus FV3000 microscope and FLUOVIEW software. Epithelial CSF1R‐Fred+ cells were enumerated using CellProfiler.^68^ 3D images were processed using Imaris (Bitplane, Zurich, Switzerland).

### Statistics

Statistical analyses were performed using GraphPad Prism 11. Differences between two groups were assessed with an unpaired two‐tailed *t*‐test or a Mann–Whitney *U*‐test. Results were deemed significant at *P < 0.05, **P < 0.01, ***P < 0.001 and****P < 0.0001.

## Author contributions

**Thi Viet Trinh Dang:** Conceptualization; data curation; formal analysis; investigation; visualization; writing – original draft; writing – review and editing. **Kevin R Gillinder:** Conceptualization; data curation; formal analysis; investigation; supervision; writing – review and editing. **Quan Nguyen:** Conceptualization; writing – review and editing. **Onkar Mulay:** Formal analysis; writing – review and editing. **Tuan Vo:** Investigation; writing – review and editing. **Ahmed M Mehdi:** Formal analysis; writing – review and editing. **Chenhao Zhou:** Formal analysis; writing – review and editing. **Andrew J Brooks:** Supervision; writing – review and editing. **Graham R Leggatt:** Supervision; writing – review and editing. **David A Hume:** Conceptualization; supervision; writing – review and editing. **Ian H Frazer:** Conceptualization; funding acquisition; supervision; writing – original draft; writing – review and editing. **Janin Chandra:** Conceptualization; funding acquisition; investigation; supervision; writing – original draft; writing – review and editing.

## Conflict of interest

The authors declare no conflict of interest.

## Supporting information


Supplementary figure 1
Supplementary figure 2
Supplementary table 1
Supplementary table 2

Video S1

Video S2

## Data Availability

The ATAC‐seq data generated in this study have been submitted to the Gene Expression Omnibus (GEO) databases under accession number GSE268544. The URL is as follows: https://www.ncbi.nlm.nih.gov/geo/query/acc.cgi?acc=GSE268544.
