# Supplementary material for: Squamous cell carcinoma is associated with reduced IL34 expression, alterations in the Langerhans cell antigen‐processing‐presentation machinery and poor patient survival
Source: Clin Transl Immunology. 2024 Nov 29;13(12):e70018. doi: 10.1002/cti2.70018 (PMC11605367; doi:10.1002/cti2.70018)
Supplement: Supplementary file 1 — Supplementary figure 1 Supplementary figure 2 Supplementary table 1 Supplementary table 2 [file CTI2-13-e70018-s002.pdf]

Supplementary figure 1

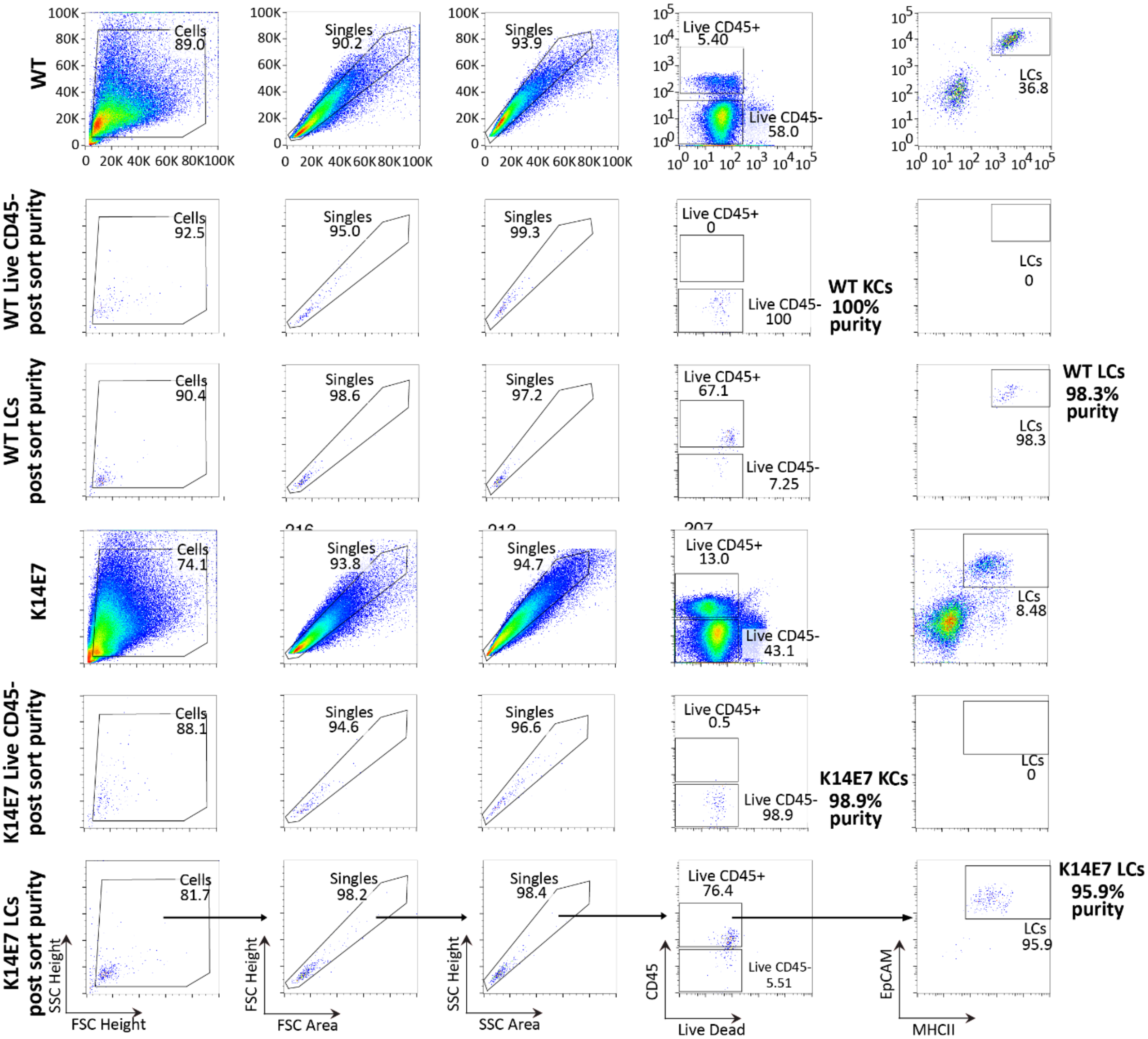

Supplementary figure 1. Gating and purity assessment of sorted KCs and LCs from one representative sorting experiment out of four conducted. In each experiment, four cell types were sorted: WT KCs, WT LCs, K14E7 KCs, and K14E7 LCs. These cells were sorted from epidermal cell obtained from either 6 WT mice or 3 K14E7 mice, which were pooled for each sample. Note: the initial experiment comprised 4 WT mice and 2 K14E7 mice.

Supplementary figure 2

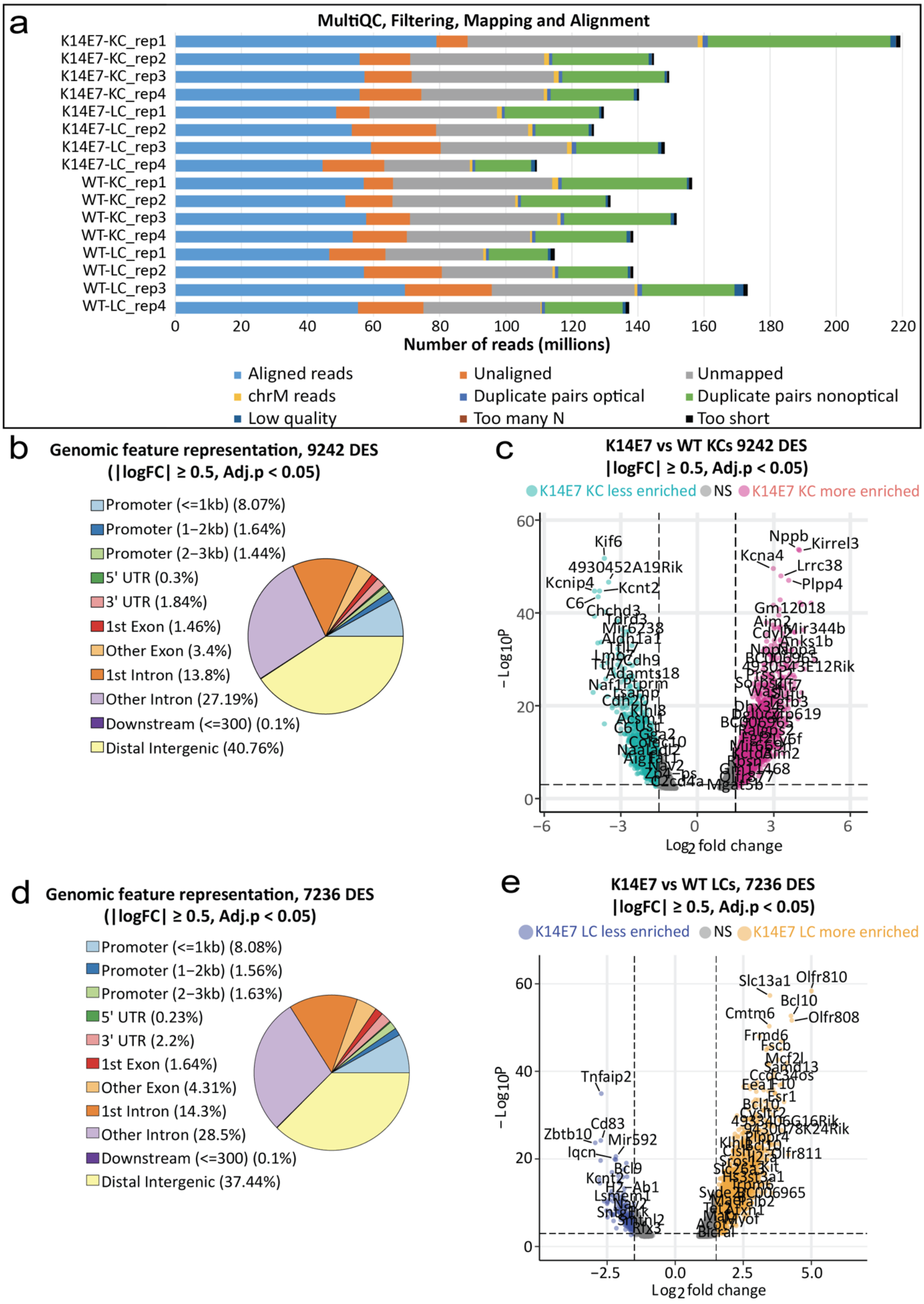

Supplementary figure 2. ATAC-seq analysis. (a) ATAC-seq reads after quality assessments. The bar chart shows the number of filtered reads (coloured) and unique aligned reads (blue) after ATAC-seq pre-analysis, which includes multiple QC, filtering, mapping, and alignment steps. 16 samples were analysed consisting of 4 cell types: K14E7 KCs, WT KCs, K14E7 LCs, and WT LCs sorted from 4 independent experiments. (b) Genomic feature representation of 9242 DES in KCs. (c) Enhanced volcano plot of annotated 9242 DES in KCs. Cutoff values (dashed line) at Adj.p = 0.05, log2FC = 1.5. (d) Genomic feature representation of 7236 DES in LCs. (e) Enhanced volcano plot of annotated 7236 DES in LCs. Cutoff values (dashed line) at Adj.p = 0.05, log2FC = 1.5.

Supplementary table 1. Antigen processing and presentation machinery (APPM) gene list with ATAC-seq and scRNA-seq outcomes.

Symbol	Gene name	ATAC-seq outcome	scRNA-seq outcome
**Antigen uptake**			
Cltc	Clathrin, heavy polypeptide (Hc)	decreased	unchanged
Clta	Clathrin, light polypeptide A (Lca)	increased	increased
Cltb	Clathrin, light polypeptide B (Lcb)	unchanged	unchanged
Scarb1	Scavenger Receptor Class B Member 1	unchanged	unchanged
Scarb2	Scavenger Receptor Class B Member 2	decreased	unchanged
Clec4e	C-Type Lectin Domain Family 4 Member E	increased	unchanged
Fcgr1	High-affinity Fc-gamma receptor	unchanged	unchanged
Fcgr2b	Low-affinity Fc-gamma receptor	increased	unchanged
Fcrl1	Fc Receptor-Like Protein 1	decreased	undetected
Fcrl5	Fc Receptor-Like Protein 5	unchanged	unchanged
Cd207	Langerin	decreased	decreased
**Processing**			
Proteasome			
Psma1	Proteasome subunit, alpha 1	increased	increased
Psma2	Proteasome subunit, alpha 2	unchanged	increased
Psma3	Proteasome subunit, alpha 3	unchanged	increased
Psma4	Proteasome subunit, alpha 4	unchanged	increased
Psma5	Proteasome subunit, alpha 5	unchanged	increased
Psma6	Proteasome subunit, alpha 6	increased	increased
Psma7	Proteasome subunit, alpha 7	unchanged	increased
Psma8	Proteasome subunit, alpha 8	unchanged	unchanged
Psmb1	Proteasome subunit, beta 1	increased	increased
Psmb2	Proteasome subunit, beta 2	unchanged	increased
Psmb3	Proteasome subunit, beta 3	increased	increased
Psmb4	Proteasome subunit, beta 4	increased	increased
Psmb5	Proteasome subunit, beta 5	unchanged	increased
Psmb6	Proteasome subunit, beta 6	unchanged	unchanged
Psmb7	Proteasome subunit, beta 7	unchanged	unchanged
Psmb8	Proteasome subunit, beta 8 (LMP7)	unchanged	unchanged
Psmb9	Proteasome subunit, beta 9 (LMP2)	unchanged	unchanged
Psmb10	Proteasome subunit, beta 10	unchanged	unchanged
Psmb11	Proteasome subunit, beta 11	unchanged	unchanged
Psmc1	Proteasome 26S subunit, ATPase 1	unchanged	unchanged
Psmc2	Proteasome 26S subunit, ATPase 2	unchanged	increased
Psmc3	Proteasome 26S subunit, ATPase 3	unchanged	increased
Psmc3ip	Proteasome 26S subunit, ATPase 3, interacting protein	unchanged	unchanged
Psmc4	Proteasome 26S subunit, ATPase, 4	unchanged	increased
Psmc5	Protease 26S subunit, ATPase 5	unchanged	increased
Psmc6	Proteasome 26S subunit, ATPase, 6	unchanged	increased
Psmd1	Proteasome 26S subunit, non-ATPase, 1	unchanged	increased
Psmd10	Proteasome 26S subunit, non-ATPase, 10	unchanged	unchanged
Psmd11	Proteasome 26S subunit, non-ATPase, 11	unchanged	unchanged
Psmd12	Proteasome 26S subunit, non-ATPase, 12	unchanged	unchanged
Psmd13	Proteasome 26S subunit, non-ATPase, 13	unchanged	increased
Psmd14	Proteasome 26S subunit, non-ATPase, 14	increased	unchanged
Psmd2	Proteasome 26S subunit, non-ATPase, 2	unchanged	increased
Psmd3	Proteasome 26S subunit, non-ATPase, 3	unchanged	increased
Psmd4	Proteasome 26S subunit, non-ATPase, 4	unchanged	unchanged
Psmd5	Proteasome 26S subunit, non-ATPase, 5	unchanged	unchanged
Psmd6	Proteasome 26S subunit, non-ATPase, 6	unchanged	increased
Psmd7	Proteasome 26S subunit, non-ATPase, 7	unchanged	increased
Psmd8	Proteasome 26S subunit, non-ATPase, 8	unchanged	increased
Psmd9	Proteasome 26S subunit, non-ATPase, 9	unchanged	unchanged
Psme1	Proteasome activator subunit 1	unchanged	increased
Psme2	Proteasome activator subunit 2	unchanged	increased
Psme3	Proteasome activator subunit 3	unchanged	unchanged
Psme4	Proteasome activator subunit 4	increased	unchanged
Psmf1	Proteasome inhibitor subunit 1	unchanged	unchanged
Psmg1	Proteasome assembly chaperone 1	unchanged	unchanged
Psmg2	Proteasome assembly chaperone 2	unchanged	unchanged
Psmg3	Proteasome assembly chaperone 3	increased	increased

Supplementary table 1 continued

Symbol	Gene name	ATAC-seq outcome	scRNA-seq outcome
Psmg4	Proteasome assembly chaperone 4	decreased	unchanged
Protease			
Ctsb	Cathepsin B	increased	increased
Ctsc	Cathepsin C	increased	unchanged
Ctse	Cathepsin E	increased	unchanged
Ctsf	Cathepsin F	decreased	unchanged
Ctsh	Cathepsin H	increased	unchanged
Ctso	Cathepsin O	unchanged	unchanged
Ctsl	Cathepsin L	unchanged	unchanged
Ctss^	Cathepsin S	increased	increased
Reductase			
Ifi30	interferon, gamma-inducible protein 30	unchanged	increased
Peptidase			
Lgmn	Legumain	increased	unchanged
Tpp1	Tripeptidyl peptidase I	unchanged	decreased
Tpp2	Tripeptidyl peptidase II	unchanged	unchanged
Nrd1	Nardilysin	unchanged	unchanged
Thop1	Thimet oligopeptidase 1	unchanged	unchanged
Erap1	Endoplasmic reticulum aminopeptidase 1	unchanged	unchanged
**Peptide loading**			
Chaperone			
Canx	Calnexin	unchanged	increased
Calr	Calreticulin	unchanged	increased
Calr3	Calreticulin 3	unchanged	unchanged
Calr4	Calreticulin 4	increased	unchanged
Pdia3	Protein disulfide isomerase associated 3	unchanged	increased
Tapbp	Tapasin, TAP binding protein	decreased	unchanged
Tapbpl	TAP binding protein-like	unchanged	unchanged
Transporter			
Tap1	Transporter associated with antigen processing 1	unchanged	increased
Tap2	Transporter associated with antigen processing 2	increased	increased
**Presentation**			
MHCI			
B2m	Beta-2 microglobulin	increased	increased
Cd1d1	CD1d1 antigen	unchanged	unchanged
Cd1d2	CD1d2 antigen	unchanged	unchanged
Mr1	Major histocompatibility complex, class I-related	unchanged	unchanged
H2-D1	Histocompatibility 2, D region locus 1	unchanged	unchanged
H2-K1	Histocompatibility 2, K1, K region	unchanged	unchanged
H2-K2	Histocompatibility 2, K region locus 2	unchanged	unchanged
H2-Ke2	H2-K region expressed gene 2	unchanged	unchanged
H2-Ke6	H2-K region expressed gene 6	decreased	unchanged
H2-M2	Histocompatibility 2, M region locus 2	decreased	decreased
H2-M3	Histocompatibility 2, M region locus 3	unchanged	increased
H2-M5	Histocompatibility 2, M region locus 5	increased	increased
H2-Q4	Histocompatibility 2, Q region locus 4	unchanged	unchanged
H2-Q5	Histocompatibility 2, Q region locus 5	unchanged	unchanged
H2-Q6	Histocompatibility 2, Q region locus 6	unchanged	unchanged
H2-Q7	Histocompatibility 2, Q region locus 7	unchanged	unchanged
H2-Q10	Histocompatibility 2, Q region locus 10	unchanged	unchanged
H2-T10	Histocompatibility 2, T region locus 10	unchanged	unchanged
H2-T22	Histocompatibility 2, T region locus 22	unchanged	increased
H2-T23	Histocompatibility 2, T region locus 23	unchanged	unchanged
H2_T24	Histocompatibility 2, T region locus 24	unchanged	unchanged
H2-Bl	Histocompatibility 2, blastocyst	unchanged	unchanged
Classical MHCII			
Cd74	CD74 antigen (invariant chain)	decreased	decreased
H2-Aa	Histocompatibility 2, class II antigen A, alpha	decreased	decreased
H2-Ab1	Histocompatibility 2, class II antigen A, beta 1	decreased	decreased
H2-Eb1	Histocompatibility 2, class II antigen E beta	decreased	decreased
H2-Eb2	Histocompatibility 2, class II antigen E beta2	unchanged	unchanged
Non-classical MHCII			
H2-DMb1	Histocompatibility 2, class II, locus Mb1	unchanged	decreased

Supplementary table 1 continued

Symbol	Gene name	ATAC-seq outcome	scRNA-seq outcome
H2-DMb2	Histocompatibility 2, class II, locus Mb2	increased	increased
H2-Dma	Histocompatibility 2, class II, locus DMa	unchanged	increased
H2-Oa	Histocompatibility 2, O region alpha locus	unchanged	unchanged
H2-Ob	Histocompatibility 2, O region beta locus	unchanged	unchanged
Ciita	Class II transactivator	increased + decreased	increased
**Co-stimulation**			
Cd80	CD80	increased	unchanged
Cd86	CD86	decreased	decreased
Cd40	CD40	unchanged	unchanged
Pvr	Poliovirus Receptor (PVR) or CD155	decreased	decreased
Cd48	Signaling lymphocytic activation molecule 2 (SLAMF2)	increased	increased
Hacvr2	Hepatitis A virus cellular receptor 2/TIM-3	unchanged	unchanged
Icosl	Inducible T cell co-stimulator ligand	increased	unchanged
Cd70	CD70	unchanged	unchanged
Timd4	T cell immunoglobulin and mucin domain containing 4	decreased	unchanged
Icam1	Intercellular adhesion molecule 1	decreased	decreased
Cd58	Lymphocyte function-associated antigen 3 (LFA-3)	unchanged	unchanged
**Co-inhibition**			
Vsir	V-set immunoregulatory receptor	decreased	unchanged
Lgals9	Lectin, galactose binding, soluble 9	increased	increased
Sirpa	Signal-regulatory protein alpha	increased + decreased	increased
Lair1	Leukocyte-associated immunoglobulin-like receptor 1	unchanged	unchanged
Clec4a1	C-type lectin domain family 4, member a1	unchanged	unchanged
Clec4a2	C-type lectin domain family 4, member a2	unchanged	unchanged
Clec4a3	C-type lectin domain family 4, member a3	increased	unchanged
Clec4a4	C-type lectin domain family 4, member a4	unchanged	unchanged
Il1r2	Interleukin 1 receptor, type II	decreased	decreased
Cd274	Programmed death-ligand 1 (PD-L1)	increased	unchanged
Pdcd1lg2	Programmed cell death 1 ligand 2 (PD-L2)	increased	unchanged
Cd276	B7 Homolog 3 (B7-H3)	decreased	undetected
Vtcn1	V-set domain containing T cell activation inhibitor 1 (B7-H4)	increased	unchanged
Lilrb2	Leukocyte immunoglobulin-like receptor, subfamily B, member 2	unchanged	unchanged
Lilrb4a	Leukocyte immunoglobulin-like receptor, subfamily B, member 4A	increased	unchanged
Lilrb4b	Leukocyte immunoglobulin-like receptor, subfamily B, member 4B	increased	unchanged
**Cytokine production**			
**Pro-inflammatory**			
Il1a	Interleukin 1 alpha	decreased	unchanged
Il1b	Interleukin 1 beta	decreased	decreased
Tnf*	Tumor Necrosis Factor	unchanged	decreased
Il6#	Interleukin 6	increased	undetected
Il12a	Interleukin 12a, p35 subunit	unchanged	unchanged
Il12b	Interleukin 12b, p40 subunit	increased + decreased	unchanged
Il23a	Interleukin 23a, p19 subunit	decreased	unchanged
Il18	Interleukin 18	increased	unchanged
Cxcl1	Chemokine (C-X-C motif) ligand 1	increased	unchanged
C3	Complement component 3	unchanged	unchanged
**Anti-inflammatory**			
Ifnb1	Interferon beta 1	increased	undetected
Il10	Interleukin 10	increased	unchanged
**Migration**			
Ccr1	Chemokine (C-C motif) receptor 1	increased	increased
Ccr5	Chemokine (C-C motif) receptor 5	increased	unchanged
Ccr7	Chemokine (C-C motif) receptor 7	increased + decreased	unchanged
Cxcr2	Chemokine (C-X-C motif) receptor 2	increased	unchanged
Cxcr3	Chemokine (C-X-C motif) receptor 3	unchanged	unchanged
Cxcr4	Chemokine (C-X-C motif) receptor 4	increased + decreased	unchanged

Supplementary table 2: Primers for RT-qPCR

mouse *Il34* forward	CTGTGCCTTATGAGGGGGTA
mouse *Il34* reverse	CGTTCTCCAGCAATGTCTGA
mouse Rps6 forward	GAAGATGATGTCCGCCAGTATG
mouse Rps6 reverse	AGTCGCTGAATCTTGGGTGC
mouse *Rpl5* forward	GTACATCGGAAGCACATCATGG
mouse *Rpl5* reverse	CTCCATCATGTCTGGAGTTACG
mouse *Eif3a* forward	CTGGAGAAGAGGACCAGCAG
mouse *Eif3a* reverse	TCCTGTCATCAGTGCGTCTC
